# USF-Net: Infrared-Visible Image Fusion via Unified Semantics and Context Modulation

**DOI:** 10.3390/s26092874

**Published:** 2026-05-04

**Authors:** Dingding Fu, Zhongguo Li, Wenbin Fan, Qi Wang

**Affiliations:** 1 School of Mechanical Engineering, Jiangsu University of Science and Technology, Zhenjiang 212100, China; 15090521845@163.com (D.F.); wqi003@126.com (Q.W.); 2 Jiangsu JBPV Intelligent Equipment Co., Ltd., Zhangjiagang 215634, China; fanwenbin@jbpv.com; 3 School of Automotive Engineering, Nantong Institute of Technology, Nantong 226001, China

**Keywords:** infrared–visible image fusion, semantic guidance, context-aware fusion, feature extraction, text-guided supervision

## Abstract

Infrared–visible image fusion aims to integrate structural details, natural appearance, and thermal target information from two source modalities, thereby improving visual perception in complex scenes. However, under challenging conditions such as low illumination, noise, low contrast, and overexposure, existing methods often struggle to stably preserve cross-modal shared features (CMSF) while effectively highlighting single-modal specific features (SMSF). In addition, the absence of real fusion labels limits effective supervised learning. To address these issues, this paper proposes a unified semantic-guided fusion network, termed USF-Net, which jointly models the shared and specific features of infrared and visible images under a unified semantic representation and dynamically adjusts the fusion strategy according to imaging contexts. Specifically, the Shared Feature Alignment and Enhancement (SFAE) module is designed to strengthen consistent modeling of common features across modalities, while the Specific Feature Reweighting Fusion (SFRF) module selectively enhances modality-specific features to achieve stable and controllable fusion. Moreover, the constructed real fusion labels are incorporated into the loss function for collaborative training. Experimental results on multiple public datasets demonstrate that USF-Net achieves superior fusion performance under diverse complex imaging conditions.

## 1. Introduction

Image fusion is an important research topic in image processing and computer vision [1]. In infrared and visible image fusion, the two source images are acquired through different imaging mechanisms. Visible images mainly record reflected scene radiation and thus provide rich texture details and natural appearance, whereas infrared images are formed from thermal radiation and can more reliably highlight salient thermal targets under nighttime, low-illumination, or complex lighting conditions [1,2]. Therefore, the objective of infrared–visible fusion is not to simply superimpose information from the two modalities, but to integrate them in a complementary manner so that the fused result can simultaneously preserve structural integrity, visual naturalness, and target saliency, while further benefiting downstream tasks such as detection and segmentation [3,4].

In practical applications, however, source images are often far from ideal. Visible images may suffer from low illumination or overexposure, while infrared images are frequently degraded by noise or reduced contrast [5]. Such challenging imaging conditions directly alter the distribution of informative content and, consequently, the preferred fusion strategy. For example, in low-light scenes, the fused image is generally expected to emphasize infrared targets while suppressing the propagation of dark noise from the visible image. In overexposed scenes, saturated bright regions should be restrained as much as possible while preserving structural layers of the scene. Under low-contrast or noisy infrared conditions, the model is further required to balance thermal target enhancement against noise suppression. Nevertheless, when existing fusion models rely only on fixed fusion rules or follow a “restore-then-fuse” pipeline, they often struggle to achieve stable optimization under a unified objective, while the overall system complexity also increases accordingly [6]. More importantly, the model must simultaneously handle two types of critical features: one is the structural and scene content shared by both modalities, which is referred to in this paper as cross-modal shared features (CMSF); the other is the information mainly contributed by a single modality and uniquely valuable to the fusion process, which is referred to as single-modal specific features (SMSF), such as thermal target saliency in infrared images and texture or appearance details in visible images. Stable alignment of CMSF and selective enhancement of SMSF are therefore central to high-quality fusion.

Most existing methods primarily depend on visual representations to distinguish and fuse CMSF and SMSF [7,8]. Although these methods can achieve reasonable performance in standard scenarios, they often lack explicit prior constraints under challenging conditions such as low illumination, noise, low contrast, and overexposure. As a result, two key questions remain insufficiently addressed: which information should be treated as shared features and consistently preserved, and which information should be regarded as modality-specific and selectively enhanced or suppressed? Since this discrimination process is largely left to implicit network learning, the model can easily exhibit unstable shared-feature alignment and imbalanced injection of modality-specific information when confronted with complex imaging conditions and cross-modal discrepancies, thereby undermining both the interpretability and robustness of the fusion results.

Recent advances in vision–language learning have shown that natural language can describe task objectives, output preferences, and constraint conditions at relatively low cost, and has gradually become an effective interaction medium for controllable vision tasks [9,10]. This line of research also opens up a new perspective for image fusion. Some recent studies have begun to introduce textual descriptions or prompts into the fusion process, yet several limitations remain. On the one hand, certain methods [11] are mainly designed for single-image restoration and do not provide an appropriate mechanism for the collaborative modeling of CMSF and SMSF in infrared–visible fusion. On the other hand, some approaches [6] rely heavily on external large models, such as GPT-4 [12], or on complex prompt engineering, which may impair training stability, reproducibility, and deployment consistency. More generally, methods driven by a single prompt and simple interaction mechanisms often find it difficult to simultaneously represent shared features, modality-specific characteristics, target preferences, and imaging conditions in a unified manner.

Meanwhile, infrared–visible image fusion has long been constrained by the lack of ground-truth fusion labels. Since no strictly standard fused image exists, existing methods usually resort to reconstruction-based losses derived from the source images as surrogate supervision. However, such supervision is inherently biased toward preserving the input content itself and cannot directly answer a more fundamental question: what constitutes a truly desirable fused result? Under complex imaging conditions, relying solely on surrogate reconstruction supervision may introduce redundant or even competing optimization objectives, making it difficult for the model to establish a consistent balance among structural fidelity, target saliency, degradation suppression, and perceptual quality. In light of this, this paper focuses on two key issues: first, how to explicitly distinguish and model CMSF and SMSF within a unified framework; second, how to construct effective supervision in the absence of ground-truth fusion labels so that the model can generate stable and controllable fusion results under different imaging contexts.

To address the above issues, this paper proposes a Unified Semantic-Guided Fusion Network (USF-Net). Specifically, a unified semantic set is first constructed to represent source-image content, cross-modal shared features (CMSF), single-modal specific features (SMSF), and the desired fusion attributes. In addition, contextual semantic descriptions are introduced to characterize the imaging conditions and task preferences associated with the input. At the architectural level, a Shared Semantic Feature Alignment Encoder (SFAE) is designed to align CMSF under semantic guidance, thereby strengthening the consistent modeling of common information across the two modalities. A Specific Semantic Feature Reweighting Fusion (SFRF) module is further developed to selectively enhance SMSF, enabling more stable and controllable injection of modality-specific information. To alleviate the lack of ground-truth fusion labels, a text-guided supervision strategy is constructed, in which textual descriptions of the expected fused results are generated in advance, and a semantic consistency loss between the predicted image and the target text is introduced to optimize the network. The main contributions of this work are summarized as follows.

A unified semantic-guided fusion network, USF-Net, is proposed for complex imaging conditions. Within a single end-to-end framework, the proposed method jointly models cross-modal shared features (CMSF) and single-modal specific features (SMSF), while introducing contextual semantic descriptions to adapt the fusion preference to different scenarios, including low illumination, noise, low contrast, and overexposure.

Two dedicated modules, namely the Shared Semantic Feature Alignment Encoder (SFAE) and the Specific Semantic Feature Reweighting Fusion (SFRF), are designed. The former semantically aligns CMSF to enhance consistent modeling of shared information, whereas the latter selectively strengthens SMSF to achieve more stable and controllable injection of modality-specific features.

A text-guided supervision mechanism is developed to address the lack of ground-truth fusion labels. By generating textual descriptions of the expected fused results in advance and computing a semantic consistency loss between the predicted image and the target text, effective supervision is provided for network optimization in the absence of true fusion labels.

Extensive experiments are conducted on multiple datasets under various challenging imaging conditions. The results demonstrate that the proposed method achieves clear improvements in both objective evaluation metrics and no-reference perceptual quality.

## 2. Related Work

### 2.1. Purely Visual Models

In recent years, end-to-end deep learning has become the dominant paradigm for visible–infrared (VIS-IR) image fusion [1,2]. Its core idea is to learn cross-modal complementary representations in the feature space and directly generate fused images, without relying on complicated traditional processing pipelines [7,8]. Most representative methods follow an encoder–fusion–decoder architecture, in which convolutional neural networks (CNNs), autoencoders, or dual-branch structures are employed to jointly preserve structural information and fine details. For example, DenseFuse enhances information flow through dense connections [7], while U2Fusion performs adaptive information selection via importance estimation [8]. Dual-stream architectures further improve detail representation and complementary feature modeling [13]. In addition, generative adversarial network (GAN)-based methods, such as DDCGAN [14], FusionGAN [15], and GANMcC [16], exploit adversarial learning to enhance the visual naturalness of fused results. Some studies have also incorporated registration [17], task-driven optimization [18], detection-oriented fusion [19], and segmentation-oriented fusion [3,4] into the fusion process to improve practical applicability. In addition, related studies have also explored thermal infrared–LiDAR or RGB–LiDAR fusion for robust perception in challenging environments [20,21].

Despite these advances, most purely visual methods still rely on fixed fusion strategies and lack explicit modeling of dynamically changing fusion preferences under complex imaging conditions. Although cascaded “restoration-then-fusion” frameworks can partially alleviate degradation effects, they are prone to introducing objective inconsistency and optimization coupling between the restoration and fusion stages.

### 2.2. Text-Guided Models

Vision–language alignment models provide a general semantic interface for controllable visual tasks. CLIP achieves image–text alignment through contrastive learning [9], while methods such as StyleCLIP further promote text-driven image editing [10]. More recently, diffusion models have significantly strengthened text-conditioned generation and controllability [22,23]. These developments also offer new opportunities for image fusion.

In the fusion domain, Text–IF introduces textual guidance for complex visual perception and processing, yet its primary focus remains single-image restoration rather than the complementary modeling required for VIS–IR fusion [6]. In particular, it does not explicitly address cross-modal complementary representation or pixel-/channel-level weight allocation for fusion. Overall, existing text-guided fusion methods still suffer from two major limitations. First, there is an inherent representational gap between semantic embeddings and visual features, making it difficult for simple interaction mechanisms to stably produce fine-grained fusion weights. Second, a single text prompt is often insufficient to simultaneously characterize shared semantics, modality-specific information, target-oriented constraints, and context-dependent fusion preferences.

### 2.3. Image Manifold-Based Domain Transform

In traditional image processing, image manifold theory treats an image as a sampled structure embedded in a high-dimensional manifold, making it possible to characterize local neighborhood relationships and guide filter design accordingly [24]. Domain transform methods proposed in [25] compress high-dimensional manifold distances into a one-dimensional space, thereby enabling efficient edge-preserving filtering. Building upon this idea, subsequent studies have integrated such mechanisms into neural networks to construct edge-aware layers.

Here, edge-aware operators refer to a class of operators that adaptively regulate the range and weight of information propagation according to local gradients, pixel differences, or neighborhood structures, so as to reduce excessive smoothing across edges. Therefore, embedding domain transform and related edge-aware modules into deep networks [26] can introduce useful priors of edge preservation and local smoothness into feature modeling, thereby improving the stable representation of structural boundaries and fine textures. Recent studies have further shown that multimodal data can establish correspondence in a shared semantic space [27], which provides a new perspective for modeling cross-modal shared features. Inspired by this idea, the proposed method employs textual semantics as guidance and combines domain-transform-based modulation with the local propagation process of visual features, so as to support semantic alignment, context modulation, and controllable fusion.

## 3. Method

### 3.1. Construction of Multiple Text Prompts and Contextual Semantic Descriptions

To overcome the limitation of a single prompt in simultaneously characterizing cross-modal shared structures, modality-complementary information, and challenging imaging contexts, multiple semantic text prompts are constructed for each input pair. Specifically, the prompts $t_{A}$ and $t_{B}$ describe the content semantics and stable structural information of each modality, respectively. The prompt $t_{AB}$ characterizes the geometric relationships and scene elements shared across modalities, thereby reinforcing the consistent modeling of common information. The prompts $t_{AS}$ and $t_{BS}$ are introduced to emphasize modality-specific advantages and complementary cues, which in turn guide the subsequent reweighted fusion process. The prompt $t_{GT}$ provides global target-level semantic constraints derived from the ideal fused result. In addition, the prompt $t_{deg}$ is used to describe the challenging conditions and task requirements of the input, and serves as a global modulation signal throughout both the shared semantic alignment stage and the specific semantic fusion stage.

**Figure 1:**
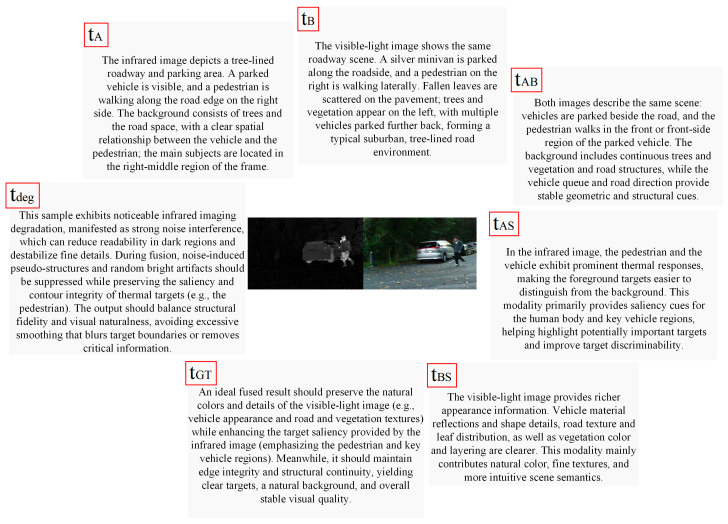
Examples of multiple text prompts customized using a noisy source image.

In this paper, the unified semantic set is generated using an offline construction mechanism based on predefined semantic templates. Specifically, according to the characteristics of the infrared–visible image fusion task, a semantic template library is first established, covering shared semantics, modality-specific semantics, target fusion attributes, and degradation contexts. Subsequently, for each input image pair, the semantic slots in the templates are instantiated according to its modality roles, scene content, complementary relationships, and degradation type, thereby generating the textual descriptions corresponding to the current sample, including $t_{A}$, $t_{B}$, $t_{AB}$, $t_{AS}$, $t_{BS}$, $t_{GT}$, and $t_{deg}$. During inference, the construction mechanism of textual descriptions remains consistent with that used in the training stage. For a given input image pair, the system instantiates the corresponding semantic descriptions according to the predefined template rules, and then feeds them into the text encoder to map them into a shared semantic space. The difference between training and inference lies in that the unified semantic set constructed during training also participates in text-supervised optimization, whereas the semantic descriptions generated during inference are only used for shared feature alignment and modality-specific feature fusion. Finally, all textual descriptions are uniformly mapped into the shared semantic space to support subsequent shared feature alignment, modality-specific feature fusion, and text-supervised optimization. Examples of the multiple text prompts are illustrated in Figure 1.

### 3.2. Model Overview

**Figure 2:**
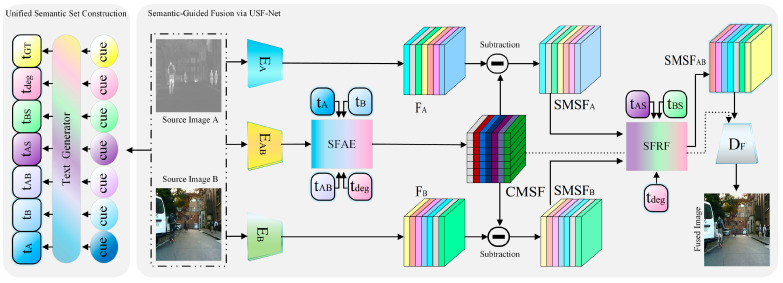
Overview of the proposed USF-Net framework. The framework consists of visual feature extractors ($E_{A}$, $E_{B}$, $E_{AB}$), textual descriptions ($t_{A},t_{B},t_{AB},t_{AS},t_{BS},t_{GT},t_{deg}$), the SFAE and SFRF modules, and an image reconstruction module. The textual descriptions are used to guide visual feature fusion in SFAE and SFRF.The solid arrows indicate the main image, feature, and textual-description flows, while the dotted arrows indicate auxiliary intermediate-feature interaction paths related to CMSF and SFRF. The shaded regions are used to separate the unified semantic set construction stage and the semantic-guided fusion stage, and the dash-dotted box is used to group the paired source images.

As illustrated in Figure 2, the proposed USF-Net takes source images A (infrared) and B (visible) as inputs. The two source images are spatially aligned and adjusted to the same size before feature extraction, so that corresponding regions can be processed on a unified spatial basis. When their original sensor resolutions are different, standard registration, resizing, or cropping can be used as preprocessing operations. This preprocessing provides a unified spatial basis for the subsequent joint feature extraction and feature fusion. The network consists of three feature extraction branches, which are used to model unimodal representations and joint representations separately. Specifically, encoder $E_{A}$ extracts the unimodal feature $F_{A}$ from image A, mainly preserving thermal responses and target saliency information. Encoder $E_{B}$ extracts the unimodal feature $F_{B}$ from image B, mainly retaining texture, edge, and appearance details. Different from these two branches, encoder $E_{AB}$ directly takes the paired input [A, B] as its input and performs joint encoding in a shared modeling space, thereby capturing cross-modal correspondences and complementary structures to obtain the joint feature $F_{AB}$. The specific backbone structures and implementation details of $E_{A}$, $E_{B}$, and $E_{AB}$ will be further described in Section 3.5.

For each input image pair, its corresponding unified semantic set is generated in advance through the offline automatic construction strategy based on predefined semantic templates, as described in Section 3.1. Specifically, the structural semantic texts $t_{A}$, $t_{B}$, and $t_{AB}$, together with the contextual semantic text $t_{deg}$, are fed into the SFAE module along with the joint feature $F_{AB}$ to obtain the cross-modal shared feature CMSF. Therefore, in USF-Net, the cross-modal shared feature CMSF is not established solely by the joint encoding process of encoder $E_{AB}$. Instead, $F_{AB}$ first provides an initial shared representation, which is then further optimized by SFAE under semantic guidance, so as to suppress inconsistent responses between modalities and strengthen stable shared structures.

After obtaining CMSF, the modality-specific features ${SMSF}_{A}$ and ${SMSF}_{B}$ are derived by subtracting CMSF from $F_{A}$ and $F_{B}$, respectively. Subsequently, ${SMSF}_{A}$ and ${SMSF}_{B}$, together with the specific semantic texts $t_{AS}$ and $t_{BS}$ and the contextual semantic text $t_{deg}$, are fed into the SFRF module to perform reweighted fusion of modality-specific information, yielding the fused specific feature ${SMSF}_{AB}$. Notably, the contextual semantic text $t_{deg}$ participates in the modulation of both the shared semantic alignment stage and the specific semantic fusion stage, enabling the fusion process to adaptively adjust according to different degradation conditions and task preferences. Finally, CMSF and ${SMSF}_{AB}$ are jointly fed into the reconstruction module $D_{F}$ to generate the final fused image.

### 3.3. Unified Semantic-Guided Shared Feature Alignment Encoder (SFAE)

**Figure 3:**
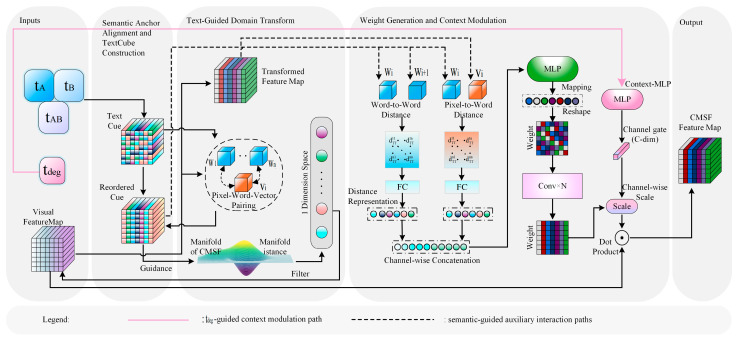
Schematic illustration of the proposed text-guided CMSF fusion module, namely SFAE. The solid arrows indicate the main feature-processing flow within SFAE, the pink lines indicate the $t_{deg}$-guided context modulation path, and the dashed lines indicate semantic-guided auxiliary interaction paths, as also shown in the legend. The shaded regions are used to visually separate the inputs, internal processing steps, and output of the module.

To strengthen the guidance of textual semantics for the extraction of cross-modal shared features (CMSF), this paper proposes a Unified Semantic-Guided Shared Feature Alignment Encoder (SFAE), as illustrated in Figure 3. The core objective of this module is to extract semantically consistent and structurally stable shared feature representations from the joint visual features under textual semantic constraints. Specifically, SFAE mainly consists of three steps. First, the textual descriptions $t_{A}$, $t_{B}$, and $t_{AB}$ are used to construct a text representation structure aligned with the visual feature space, thereby establishing explicit correspondences between textual semantics and visual features. Second, through semantic reordering and text-guided domain transformation, the semantic structure is introduced into the visual feature propagation process, so as to enhance the semantic consistency of the shared features. Finally, the visual–text distance, text–text distance, and contextual semantics $t_{deg}$ are jointly used to generate and modulate the weights, yielding the final CMSF feature map. Based on the above process, SFAE can collaboratively model cross-modal shared information from three aspects, namely semantic alignment, propagation constraint, and context modulation.

#### 3.3.1. Semantic Anchor Alignment and TextCube Construction

To achieve structured interaction between joint visual features and textual semantics, this paper constructs a TextCube whose dimensions are consistent with the visual feature map based on the three semantic descriptions $t_{A}$, $t_{B}$, and $t_{AB}$. Let the size of the joint visual feature map V be H × W × C, where H, W, and C denote the height, width, and number of channels of the feature map, respectively.

First, the three semantic texts are linearly projected through multilayer perceptrons (MLPs), so that their embedding lengths correspond to the three dimensions of the visual feature map. Specifically, $t_{A}$ is mapped to the height dimension H, $t_{B}$ is mapped to the width dimension W, and $t_{AB}$ is mapped to the channel dimension C. Therefore, the mapped semantic vectors are denoted as $t_{A}^{H}\in R^{H}$, $t_{B}^{W}\in R^{W}$, and $t_{AB}^{C}\in R^{C}$, respectively.

Then, the TextCube T is constructed based on the three mapped semantic vectors. Specifically, the outer product of $t_{A}^{H}$ and $t_{B}^{W}$ is first used to generate a two-dimensional spatial semantic distribution. This two-dimensional semantic distribution is then reshaped into a spatial semantic tensor of size H × W × 1, and further interacted with the channel semantic vector $t_{AB}^{C}$, thereby producing the final TextCube representation. The core construction process is formulated as follows:

(1) $$T=\zeta \left(\zeta \left(t_{A}^{H}⊗{t_{B}^{W}}^{T},\left[HW,1\right]\right)⊗t_{AB}^{C}\right),\left[H,W,C\right],$$

Here, ⊗ denotes matrix multiplication, and ζ denotes the tensor reshape operation. In this way, a TextCube explicitly aligned with the spatial scale of the visual feature map is constructed, allowing semantic information to participate jointly in both spatial-dimension and channel-dimension modeling. This provides finer-grained semantic guidance for subsequent cross-modal feature fusion.

On this basis, to achieve stable alignment between textual semantics and visual features, a similarity-based semantic reordering strategy is further applied to the TextCube. Let $v\left(i,j\right)$ denote the feature vector at position $\left(i,j\right)$ in the visual feature map V, and let $w\left(i,j\right)$ denote the corresponding word vector in the TextCube. For each visual feature $v\left(i,j\right)$, its similarity to all word vectors in the TextCube is computed, and the most similar semantic position ($x^{*},y^{*}$) is selected:

(2) $$w\left(i,j\right)\leftarrow w\left(x^{*},y^{*}\right),\;s.t.\;\left(x^{*},y^{*}\right)=arg\underset{\left(x,y\right)}{max}\;sim\left(v\left(i,j\right),w\left(x,y\right)\right)\forall \left(i,j\right),$$

Here, $sim\left(\cdot \right)$ denotes the cosine similarity function, and argmax returns the coordinate corresponding to the maximum similarity. Through this reordering process, the semantic representation in the TextCube can be dynamically adjusted according to the visual features, thereby improving the consistency between the text features at each spatial location and their corresponding visual features.

In summary, this step first constructs a semantic representation structure aligned with the visual feature space, and then establishes more stable pixel–semantic correspondences through similarity-based reordering. This provides a unified semantic basis for the subsequent propagation and modulation of shared features.

#### 3.3.2. Text-Guided Domain Transform

This step aims to constrain the propagation of visual features by using the semantic structure provided by text, thereby achieving semantically consistent feature modulation. For the reordered TextCube T, the distances between adjacent word vectors can characterize the degree of semantic variation. Based on this observation, textual semantics are used to guide a domain transform on the visual feature map V. Specifically, the semantic distances between neighboring word vectors in the TextCube are first computed, and the high-dimensional semantic distances are compressed into a one-dimensional equidistant distance d through domain transformation. Subsequently, the visual features are smoothed by a recursive filter, whose core form is given by

(3) $$V\left[n\right]=\left(1-a^{d}\right)T\left[n\right]+a^{d}T\left[n-1\right],\;n>1,$$

Here, $a\in \left(0,1\right)$ is the feedback coefficient, and d denotes the semantic distance. Through the above operation, the domain-transformed visual feature $V_{dt}$ is obtained.

Different from conventional domain transform methods that are driven by visual gradients, the proposed text-guided domain transform modulates the feature propagation range by leveraging textual semantic information, thereby enhancing the semantic consistency between cross-modal features.

From the perspective of the overall workflow, this step explicitly introduces the semantic structure into the visual feature propagation process, so that the smoothing and propagation of shared features no longer depend solely on local visual variations, but are also constrained by textual semantics.

#### 3.3.3. Weight Generation and Context Modulation

First, the cosine distance DTV between the matched word vector $w\left(i,j\right)$ and the visual feature vector $v\left(i,j\right)$ is computed. In addition, the distance $D_{TT}$ between adjacent word vectors is also calculated. The two distance measures, $D_{TV}$ and $D_{TT}$, are then separately encoded by multilayer perceptrons (MLPs) to generate two types of spatial response weights, denoted as $W_{TV}^{\prime }$ and $W_{TT}^{\prime }$, respectively. These two weights are combined by element-wise summation to obtain the fused weight vector $W_{F}^{\prime }$, which is further reshaped into a two-dimensional weight matrix $W_{F}\in R^{H\times W}$. Then, Conv × N is adopted as a lightweight refinement operation to improve the local consistency of $W_{F}$. Finally, $V_{dt}$ is multiplied element-wise by $W_{F}$ to produce the preliminary representation of the shared semantic feature:

(4) $$V_{CMSF}^{\left(0\right)}=V_{dt}⊙W_{F},$$

On this basis, the contextual semantic vector $t_{deg}$ is introduced as a context modulation signal to further refine the shared features through channel-wise gating, yielding the final CMSF feature map. The channel weights are generated by a mapping function, which can be expressed as

(5) $$V_{CMSF}\left(c,i,j\right)=g_{deg}\left(c\right).V_{CMSF}^{\left(0\right)}\left(c,i,j\right),\;c=1,\ldots ,C,$$

where c denotes the channel index in the feature map, and C denotes the total number of channels of the corresponding feature map. In our implementation, the channel dimension of the main feature representation is set to 64; therefore, C is set to 64 in this expression. The coefficient $g_{deg}\left(c\right)$ denotes the context modulation coefficient for the c-th channel.

Unlike conventional fixed fusion strategies, the proposed mechanism jointly exploits distance-driven spatial weighting and context-aware channel modulation, enabling the shared semantic features to be adaptively adjusted according to the textual semantic structure, thereby improving the stability of cross-modal feature alignment.

In summary, SFAE first establishes explicit correspondences between textual semantics and visual features through semantic structure construction. Then, text-guided domain transformation is employed to constrain the propagation of shared features. Finally, spatial weighting and contextual modulation are combined to generate the final CMSF representation, thereby achieving stable alignment and enhancement of cross-modal shared information.

### 3.4. Unified Semantic-Guided Specific Feature Reweighting Fusion (SFRF)

**Figure 4:**
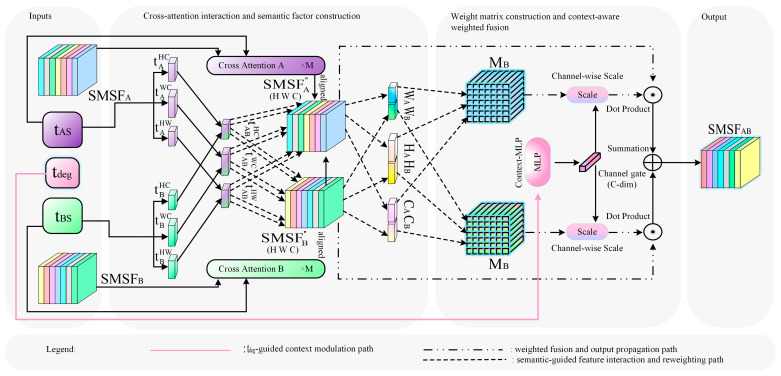
Schematic illustration of the proposed text-guided SMSF fusion module, namely SFRF. The solid arrows indicate the main feature-processing flow within SFRF. The pink lines indicate the $t_{deg}$-guided context modulation path, the dash-dotted arrows indicate the weighted fusion and output propagation path, and the dashed arrows indicate the semantic-guided feature interaction and reweighting path, as also shown in the legend. Different colors are used to distinguish modality-specific features, semantic factors, and intermediate feature representations for clearer visualization. The shaded regions are used to visually separate the inputs, cross-attention interaction and semantic factor construction, weight matrix construction and context-aware weighted fusion, and output of the module.

To effectively exploit textual semantics for guiding the fusion of single-modal specific features (SMSF), this paper proposes a Unified Semantic-Guided Specific Feature Reweighting Fusion (SFRF) module, as illustrated in Figure 4. The module takes as input the visually pre-aligned features $SMSF_{A}^{\prime \prime }$ and $SMSF_{B}^{\prime \prime }$, obtained through cross-attention [28], together with the textual features $t_{AS}$ and $t_{BS}$. Its objective is to achieve adaptive reweighted fusion of the specific information from the two modalities while preserving modality-specific advantages.

Specifically, SFRF mainly consists of two steps. First, the textual features are mapped into semantic vectors corresponding to different dimensions of the visual features, and text–visual interaction is performed to extract structural cues that can reflect modality preferences. Second, modality weight matrices are constructed according to these semantic interaction results, and the contextual semantic vector $t_{deg}$ is further introduced for channel-wise modulation. In this way, the contribution ratios of the two modalities can be dynamically adjusted under different imaging contexts, and the fused specific feature representation $SMSF_{AB}$ is finally generated. Therefore, the overall process of SFRF can be summarized as semantic interaction, weight construction, context modulation, and specific-information fusion.

#### 3.4.1. Text–Visual Interaction and Edge Vectors

To enable effective interaction between textual semantics and visual features, the semantic text representations are first mapped by multilayer perceptrons (MLPs) to generate semantic vectors corresponding to different dimensions of the visual features. Through this process, textual semantics are encoded into multi-scale semantic representations, allowing them to interact separately with the spatial and channel dimensions of the visual features.

Subsequently, the generated semantic vectors are combined with the unimodal feature SMSF” through cross-dimensional interaction, so that textual semantics can modulate the visual features. Taking the interaction between the spatial semantic vector and the visual feature as an example, the core formulation is given by

(6) $$v_{A}^{c}=t_{A}^{HW}⊗\zeta \left(SMSF_{A}^{\prime \prime },\left[HW,C\right]\right),$$

Here, $\zeta \left(\cdot \right)$ denotes the tensor reshape operation, and ⊗ denotes matrix multiplication. The resulting $v_{A}^{c}$ is an edge vector of length C.

Through multi-dimensional semantic mapping and cross-dimensional interaction, textual semantics can jointly modulate visual features at both the spatial and channel levels. This enables a structured fusion mechanism that differs from conventional global text embeddings and strengthens the semantic association between cross-modal features.

In summary, this step maps textual semantics into multidimensional semantic vectors that can structurally interact with specific visual features, and further extracts edge and structural cues reflecting modality preferences.

#### 3.4.2. Weight Matrix Construction and Context Modulation

Using the edge semantic vectors obtained in the previous subsection, they are projected onto the same spatial and channel dimensions as the visual features, thereby producing the weight matrices $M_{A}$ and $M_{B}$. To further regulate the fusion weights, the contextual semantic vector $t_{deg}$ is introduced for context modulation. This vector is used to characterize the overall properties of the current semantic context, allowing the fusion process to adaptively adjust according to different contextual conditions. Specifically, a channel-wise modulation coefficient is generated through a mapping function:

(7) $$g_{deg}=f\left(t_{deg}\right),$$

Here, $g_{deg}$ denotes the channel scaling vector. By applying this coefficient to the weight matrices in a channel-wise manner, the context-modulated weights ${\hat{M}}_{A}$ and ${\hat{M}}_{B}$ are obtained.

Subsequently, the two modality-specific features are fused by element-wise weighted summation under the guidance of the semantic weights, with the core formulation given by

(8) $$SMSF_{AB}={\hat{M}}_{A}⊙SMSF_{A}^{\prime \prime }+{\hat{M}}_{B}⊙SMSF_{B}^{\prime \prime },$$

where ⊙ denotes element-wise multiplication.

Different from fixed fusion strategies, this module dynamically adjusts the contributions of the two modalities through semantics-driven context modulation, thereby achieving stable and adaptive feature reweighting.

In summary, SFRF first extracts semantic cues reflecting modality preferences through text–visual interaction. Then, modality weight matrices are constructed and further modulated by contextual information, thereby achieving stable and adaptive reweighted fusion of modality-specific features.

### 3.5. Visual Feature Extraction and Reconstruction

The visual branch of USF-Net consists of three feature extractors, $E_{A}$, $E_{B}$, and $E_{AB}$, together with a reconstruction module $D_{F}$. The unimodal feature extractors $E_{A}$ and $E_{B}$ are implemented based on invertible neural networks (INNs) [29,30] to reduce information loss during feature encoding, thereby obtaining stable unimodal feature representations. The cross-modal feature extractor $E_{AB}$ is built upon RetNet [31], which jointly models the input modalities [A, B] to extract the shared feature $F_{AB}$, while also providing multi-scale features for the subsequent semantic interaction and context modulation modules, namely SFAE and SFRF. Finally, the reconstruction module $D_{F}$ adopts a lightweight U-Net-like architecture to map the shared feature CMSF and the specific feature SMSF back to the image space, thereby generating the fused image ${\hat{I}}_{F}$.

### 3.6. Multi-Stage Training and Loss Functions

To reduce the difficulty of cross-modal training and improve the stability of semantic alignment, USF-Net adopts a two-stage training strategy.

#### 3.6.1. Stage I: Pure Visual Pretraining

In the first stage, the semantic modules SFAE and SFRF are deactivated, and only the visual branch and reconstruction module are retained, allowing the network to first learn a stable visual fusion representation. The loss function is defined as

(9) $$L^{\left(1\right)}=\alpha_{0}L_{MSE}+\beta_{0}L_{SSIM}+\gamma_{0}L_{L1},$$

Here, $L_{MSE}$ (mean squared error), $L_{SSIM}$ (structural similarity loss), and $L_{L1}$ (L1 loss) are used to minimize the pixel-level discrepancy between the fused image and the source images, while improving structural consistency and detail preservation. The coefficients $\alpha_{0}$, $\beta_{0}$, and $\gamma_{0}$ denote the baseline weighting factors. Among them, $\alpha_{0}$ serves as the main optimization anchor for the pixel-level reconstruction term, ensuring basic intensity consistency and training stability of the fused results. $\beta_{0}$ is used to constrain overall structural fidelity, while $\gamma_{0}$ is set slightly higher than $\beta_{0}$ to further strengthen local detail preservation and edge retention.

#### 3.6.2. Stage II: Joint Text–Visual Fine-Tuning

After the first stage converges, the semantic modules SFAE and SFRF, together with the text loss, are introduced to perform end-to-end fine-tuning of the whole network. The overall loss is defined as

(10) $$L^{\left(2\right)}=\alpha_{0}L_{MSE}+\beta_{0}L_{SSIM}+\gamma_{0}L_{L1}+\eta_{0}L_{TEXT},$$

where $L_{TEXT}$ denotes the proposed text-guided loss.

To achieve semantics-driven adaptive optimization, the contextual semantic vector $t_{deg}$ is further introduced to modulate the loss weights. Specifically, a weight scaling coefficient is generated through a mapping function:

(11) $$s_{deg}=clip\left(\sigma \left({MLP}_{deg}\left(t_{deg}\right)\right)\right),$$

The baseline weights are then adjusted as

(12) $$\alpha =s_{\alpha }\alpha_{0},\beta =s_{\beta }\beta_{0},\gamma =s_{\gamma }\gamma_{0},\eta =s_{\eta }\eta_{0},$$

This mechanism dynamically adjusts the importance of each loss term according to the contextual semantics, thereby stabilizing the optimization process under different challenging conditions.

The text loss is used to constrain the consistency between the fused image and multiple semantic descriptions:

(13) $$L_{TEXT}=\xi \left(t_{F},t_{A}\right)+\xi \left(t_{F},t_{B}\right)+\xi \left(t_{F},t_{GT}\right),$$

where $t_{A}$, $t_{B}$, and $t_{GT}$ denote the text embeddings, $t_{F}$ denotes the embedding of the fused image, and $\xi \left(\cdot \right)$ represents the semantic distance function. This loss imposes semantic-level constraints on the fusion result, enabling the fused image to preserve multi-source information while approaching the desired semantic expression.

## 4. Experimental Results and Analysis

### 4.1. Experimental Settings

The proposed method was implemented in the PyTorch 2.9.0 framework and trained on an NVIDIA GeForce RTX 4090 GPU. The total number of training epochs was set to 150, including 100 epochs for the training stage and 50 epochs for the fine-tuning stage. The batch size was 16, and the Adam optimizer was adopted. The initial learning rate was set to 1 × 10^−4^ and decayed by a factor of 0.5 every 20 epochs. The encoder $E_{AB}$ used in SFRF consists of four RetNet blocks, each with six attention heads and a feature dimension of 64. The baseline weighting coefficients $\alpha_{0}$, $\beta_{0}$, $\gamma_{0}$, and $\eta_{0}$ were set to 1, 0.5, 0.6, and 0.5, respectively.

### 4.2. Benchmark Settings

#### 4.2.1. Evaluation Metrics

To comprehensively evaluate the performance of the proposed method, this paper adopts an evaluation strategy that combines reference-based fusion metrics and no-reference perceptual metrics. The reference-based fusion metrics include entropy (EN) [1], standard deviation (SD) [1], spatial frequency (SF) [1], sum of the correlations of differences (SCD) [32], visual information fidelity (VIF) [33], and gradient-based fusion quality metric $Q^{AB/F}$ [34]. Among them, EN is used to measure the amount of information contained in the fused image. SD reflects the degree of contrast variation and the overall gray-level fluctuation of the image. SF is adopted to evaluate the spatial activity and detail richness of the fused image. SCD measures the extent to which the fused result preserves complementary information from the two source images. VIF is used to assess the visual information fidelity of the fused image with respect to the source images. $Q^{AB/F}$ reflects the transfer quality of edge and gradient information from the source images to the fused result. Unless otherwise specified, larger values of these metrics generally indicate better fusion performance.

In addition, to further evaluate perceptual quality and naturalness under complex imaging conditions, four no-reference perceptual metrics are introduced, including NIQE [35], BRISQUE [36], MUSIQ [37], and CLIP-IQA [38]. Among them, NIQE and BRISQUE are used to measure perceptual distortion and image naturalness, where lower values indicate better image quality. MUSIQ is adopted to evaluate multi-scale perceptual image quality, while CLIP-IQA measures the consistency between semantic preference and perceptual quality. For these two metrics, larger values indicate better perceptual quality.

Considering that this study focuses on infrared–visible image fusion under complex degradation conditions, the evaluation design should not only emphasize the retention of information from the source images, but also consider perceptual quality, visual naturalness, and scene adaptability after fusion. Therefore, instead of exhaustively reporting all possible metrics, this paper selects a representative set of metrics from several complementary aspects, including information content, contrast and texture, visual fidelity, perceptual distortion, and semantic-aware perceptual quality, so as to achieve a balance between evaluation completeness and readability. In general, the adopted evaluation system is not intended to simply stack more metrics, but to provide a comprehensive assessment of the fusion results from the perspectives of information fidelity, structural consistency, visual quality, and semantic perception.

#### 4.2.2. Datasets

To verify the generalization ability of the proposed model under different scenes and imaging conditions, four commonly used infrared–visible fusion datasets were adopted, namely MSRS [5], MFNet [39], RoadScene [8], and LLVIP [40]. These datasets cover road scenes, urban environments, and nighttime pedestrian scenarios, and include a variety of imaging degradations, such as low illumination, overexposure, insufficient contrast, and infrared noise.

After merging the four datasets, the image pairs were divided in a stratified manner according to data source and scene category, resulting in 3618 pairs for training and 1135 pairs for testing. During evaluation, results on each subset are reported separately, together with the overall average. A two-stage training strategy was adopted. In the first stage, pure visual pretraining was conducted on the entire training set. In the second stage, 1278 image pairs were sampled from the training set in a stratified manner to load the unified semantic set for semantic–visual collaborative fine-tuning, so as to reduce the cost of semantic construction and avoid distribution shift.

#### 4.2.3. Definition of Experimental Settings

To ensure a fair and consistent comparison, two experimental settings were defined in this work, namely semantics-off and semantics-on. The only difference between the two settings lies in whether the unified semantic set and its associated mechanisms are enabled. All other components, including the network architecture, training strategy, data partition, and hyperparameter settings, remain unchanged to guarantee comparability.

In the semantics-off setting, the semantic branches of SFAE and SFRF, the text-guided loss, and the contextual semantic modulation are all disabled. Only the visual backbone and the reconstruction network are retained, so as to evaluate the performance of the pure visual baseline.

In the semantics-on setting, the unified semantic set and the text-guided loss are enabled, and contextual semantic modulation is injected into SFAE and SFRF. This setting is used to report the performance of the complete model.

### 4.3. Comparison with State-of-the-Art Methods

To validate the effectiveness of the proposed method, eight representative fusion models from recent years were selected for comparison, including ReCoNet [41], PIAFusion [5], U2Fusion [8], MetaFusion [42], SemLA [43], MRFS [44], SAGE [45], and DCEvo [46].

#### 4.3.1. VIS-IR Fusion Under the Semantics-Off Setting

**Figure 5:**
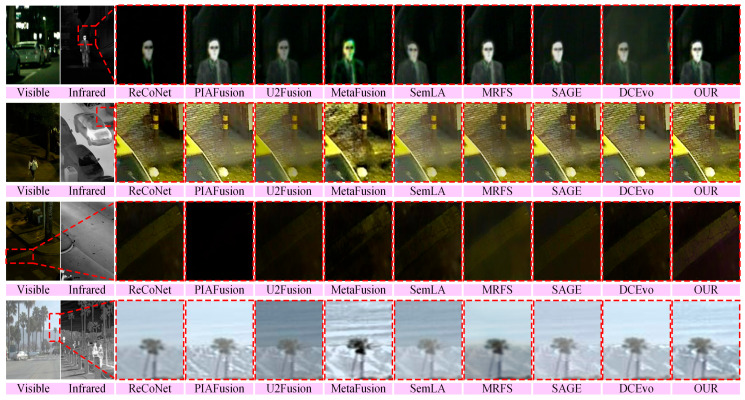
From top to bottom, qualitative comparison on samples from the MSRS, LLVIP, and RoadScene datasets. From left to right, the first two columns show the infrared and visible source images, respectively, and the remaining columns present the fusion results of different methods.

To ensure a fair comparison, only the fusion performance without semantic guidance is considered in this setting. Accordingly, USF-Net does not introduce any additional semantic information. The corresponding quantitative results are reported in Table 1, while the qualitative comparisons are presented in Figure 5.

As shown in Figure 5, compared with the competing methods, OURS produces clearer salient thermal targets, achieves a better balance between highlight suppression and dark-detail preservation, and maintains colors and brightness levels closer to the visible-image perception in daytime scenes. By contrast, some methods suffer from missing dark textures, over-enhanced bright regions, or color deviation, which weakens local structural readability. These observations are in agreement with the quantitative improvements in VIF and $Q^{AB/F}$.

Table 1 reports the quantitative comparison results of five objective metrics on the MSRS, LLVIP, and RoadScene datasets under the semantics-off setting. Overall, the proposed method demonstrates a more stable comprehensive advantage in cross-dataset evaluation, with particularly consistent superiority on metrics reflecting structural fidelity and edge transfer, such as VIF and $Q^{AB/F}$. On the MSRS dataset, the proposed method achieves the best SCD, SD, VIF, and $Q^{AB/F}$ values, indicating stronger structural consistency, information preservation, and gradient transfer ability. On the LLVIP dataset, it obtains the best results on all reported metrics, demonstrating better information content and fusion quality in nighttime pedestrian scenes. On the RoadScene dataset, the proposed method achieves the best EN, VIF, and $Q^{AB/F}$ values, while remaining competitive on SCD and SD. These cross-scene consistent results further verify the generalization ability and robustness of the proposed method under different imaging conditions and scene distributions.

#### 4.3.2. VIS-IR Fusion Under the Semantics-On Setting

**Figure 6:**
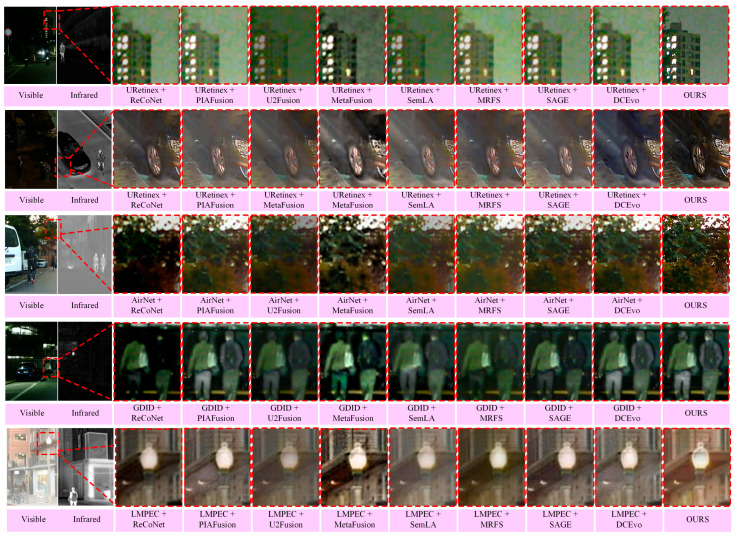
Qualitative comparison under challenging imaging conditions. From top to bottom, the examples correspond to low-light visible images from MSRS, low-light visible images from LLVIP, low-contrast infrared images from MFNet, noisy infrared images from DN-MSRS, and overexposed visible images from RoadScene, respectively.

In real-world scenarios, source images are often accompanied by challenging degradations, such as low illumination, noise, low contrast, and local overexposure, which can easily lead to brightness imbalance, detail distortion, and artifact amplification in the fused results. To enable a fairer and more interpretable comparison under such complex inputs, the representative fusion methods are combined with their corresponding image restoration models (denoted as enhance/fusion). Specifically, URetinex [47] is adopted for low-light enhancement, AirNet [48] for low-contrast restoration, GDID [49] for denoising, and LMPEC [50] for overexposure correction. In contrast, USF-Net performs adaptive fusion across all five challenging scenarios using a unified model with a fixed parameter configuration, demonstrating stronger universality and better deployment consistency. The corresponding quantitative results are reported in Table 2 and Table 3, while the qualitative comparisons are presented in Figure 6.

As shown in Figure 6, the proposed method achieves a more stable trade-off among thermal target saliency, structural detail preservation, and the naturalness of color and brightness. Specifically, under low-light conditions, it enhances visibility while suppressing local overexposure, meanwhile preserving clear target contours and background linear structures. For low-contrast and noisy infrared inputs, the fused results are cleaner and exhibit better edge separation, thereby reducing structural blurring caused by noise injection and excessive smoothing. In overexposed visible-light scenes, the proposed method more effectively suppresses highlighted regions while maintaining overall brightness and color consistency as much as possible. In contrast, some enhance/fusion combinations still suffer from globally dark appearance and color bias, missing bright-region details, or residual noise in certain samples, which degrades local structural readability.

Table 2 and Table 3 report the quantitative comparison results under five challenging imaging conditions. Overall, the proposed method achieves better performance on most datasets, with its advantages mainly reflected in no-reference perceptual quality metrics as well as structure- and contrast-related indicators. In the low-light scenarios of MSRS and LLVIP, the proposed method simultaneously improves information content and contrast while reducing NIQE, indicating better perceptual quality under degraded illumination. On MFNet and DN-MSRS, it maintains leading performance on metrics such as MUSIQ and SD, while also achieving lower NIQE, suggesting a better trade-off between detail enhancement and noise suppression. In the overexposed scenario of RoadScene, the proposed method obtains the best NIQE and BRISQUE scores, demonstrating more effective distortion suppression. Although certain individual metrics, such as SF, are slightly higher for some competing methods, these gains do not translate into an overall advantage in perceptual quality. This result indicates that the proposed method delivers more consistent quality improvement across diverse challenging distributions.

### 4.4. Efficiency Comparison

To further verify the practical deployment capability of the proposed method, this paper adopts the number of parameters (Params) and floating-point operations (FLOPs) to measure model complexity, and evaluates computational efficiency by calculating the frames per second (FPS) on the MSRS, LLVIP, and RoadScene datasets. The number of parameters reflects the storage cost of the model, while FLOPs indicate its computational demand. Together, these two indicators characterize the overall model complexity. A higher FPS indicates higher running efficiency. Under the same hardware environment, the proposed method is further compared with the aforementioned representative methods in terms of efficiency, and the results are reported in Table 4.

As shown in Table 4, the Params and FLOPs of the proposed method are only higher than those of a few lightweight methods, and its overall complexity ranks third among all compared methods, remaining at a relatively low level. In terms of running efficiency, the proposed method ranks second in FPS on the MSRS and LLVIP datasets, and third on the RoadScene dataset, maintaining first-tier efficiency overall and clearly outperforming most competing methods. These results demonstrate that the proposed method can maintain controllable computational cost while ensuring strong fusion performance. Therefore, it achieves a favorable balance among fusion performance, model complexity, and running efficiency, showing certain potential for practical deployment.

### 4.5. Ablation Study

**Figure 7:**
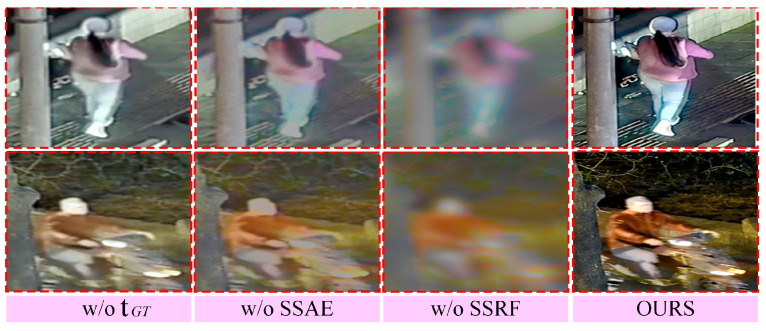
Qualitative comparison of ablation results for the target-level semantic prompt and key modules.

A series of systematic ablation experiments were conducted to comprehensively evaluate the effectiveness of the proposed Shared Semantic Feature Alignment Encoder (SFAE), Specific Semantic Feature Reweighting Fusion (SFRF) module, and the global target-level semantic constraints provided by $t_{GT}$. The corresponding quantitative results are reported in Table 5, while the qualitative comparisons are presented in Figure 7.

As shown in Table 5 and Figure 7, when the Shared Semantic Feature Alignment Encoder (SFAE) or the Specific Semantic Feature Reweighting Fusion (SFRF) module is removed, the fused results exhibit simultaneous degradation in both structural consistency and complementary information representation. This indicates that shared-feature alignment and specific-feature reweighting constitute two complementary and indispensable stages in the proposed framework. In addition, when $t_{GT}$ is removed, the model lacks global target-level semantic constraints derived from the ideal fused result and becomes more prone to fusion-target drift, leading to declines in overall visual quality as well as in structural and semantic stability.

## 5. Conclusions

In this study, we propose a novel Unified Semantic-Guided Fusion Network (USF-Net) to improve the stability and perceptual quality of infrared–visible image fusion under challenging imaging conditions. To accommodate the variations in information distribution and fusion requirements arising in scenarios such as low illumination, noise, low contrast, and overexposure, we construct a unified semantic representation together with contextual semantic descriptions, and incorporate them throughout the key stages of the fusion framework. This mechanism not only enhances the consistent modeling of cross-modal shared features (CMSF), but also provides semantic support for adapting the fusion strategy under different challenging conditions. Furthermore, we design a Shared Semantic Feature Alignment Encoder (SFAE) and a Specific Semantic Feature Reweighting Fusion (SFRF) module to improve the alignment of CMSF and the enhancement of single-modal specific features (SMSF), respectively. Meanwhile, to address the lack of ground-truth fusion labels, the constructed real fusion labels and text-guided supervision are jointly incorporated into the loss function to improve optimization under complex conditions. Experimental results on multiple datasets and diverse challenging scenarios demonstrate that the proposed method achieves favorable overall performance. These results indicate that USF-Net has strong application potential for infrared–visible image fusion in complex environments and provides a new perspective for semantic-guided multimodal fusion.

## Figures and Tables

**Table 1:** Quantitative evaluation results on the MSRS, LLVIP, and RoadScene datasets under the semantics-off setting; red indicates the best result, and blue indicates the second-best result.

Dataset	Methods	SCD	SD	EN	VIF	$Q^{AB/F}$
MSRS	ReCoNet	1.191	44.374	5.052	0.433	0.367
	PIAFusion	1.522	41.953	6.746	0.925	0.575
	U2Fusion	1.182	23.541	5.246	0.506	0.372
	MetaFusion	1.486	39.432	6.368	0.726	0.478
	SemLA	1.254	30.518	5.953	0.664	0.458
	MRFS	1.431	39.843	6.551	0.723	0.489
	SAGE	1.733	44.912	6.871	1.024	0.643
	DCEvo	1.756	45.632	6.901	1.101	0.675
	OURS	1.802	46.732	6.886	1.171	0.693
Dataset	Methods	SCD	SD	EN	VIF	$Q^{AB/F}$
LLVIP	ReCoNet	1.345	41.234	5.514	0.513	0.364
	PIAFusion	1.323	44.853	6.523	0.882	0.465
	U2Fusion	0.757	23.614	5.972	0.552	0.341
	MetaFusion	1.317	42.446	6.823	0.833	0.493
	SemLA	1.036	27.984	5.981	0.631	0.364
	MRFS	1.123	35.485	6.263	0.581	0.395
	SAGE	1.581	47.972	7.124	0.982	0.585
	DCEvo	1.664	49.768	7.453	1.113	0.653
	OURS	1.702	50.621	7.542	1.321	0.687
Dataset	Methods	SCD	SD	EN	VIF	$Q^{AB/F}$
RoadScene	ReCoNet	1.589	37.581	6.822	0.504	0.354
	PIAFusion	1.586	49.283	6.975	0.701	0.453
	U2Fusion	1.498	30.969	6.739	0.513	0.467
	MetaFusion	1.581	51.643	7.223	0.512	0.468
	SemLA	1.248	31.869	6.548	0.503	0.438
	MRFS	1.399	40.874	6.947	0.501	0.431
	SAGE	1.758	51.637	7.073	0.658	0.497
	DCEvo	1.642	49.833	7.468	0.801	0.611
	OURS	1.651	50.816	7.476	0.853	0.621

**Table 2:** Quantitative comparison results under low-light visible conditions on the MSRS and LLVIP datasets; “eir.” denotes the corresponding existing image restoration preprocessing method used for each degradation setting, red indicates the best result, and blue indicates the second-best result.

Method	MSRS Dataset			LLVIP Dataset		
	CLIP-IQA	EN	NIQE	EN	NIQE	MUSIQ
eir. + ReCoNet	0.117	7.216	5.769	7.109	4.695	44.187
eir. + PLAFusion	0.123	7.082	3.781	7.332	3.986	48.255
eir. + U2Fusion	0.127	6.724	3.997	7.439	3.969	48.481
eir. + MetaFusion	0.106	7.307	3.584	7.495	3.722	49.628
eir. + SemLA	0.113	6.861	3.944	7.214	4.184	46.053
eir. + MRFS	0.121	7.051	3.822	7.281	4.061	47.241
eir. + SAGE	0.131	7.275	3.563	7.552	3.683	50.356
eir. + DCEvo	0.132	7.292	3.521	7.583	3.621	50.836
OURS	0.134	7.301	3.47 8	7.624	3.541	51.21 7

**Table 3:** Quantitative comparison results under low-contrast infrared, noisy infrared, and overexposed visible conditions on the MFNet, DN-MSRS, and RoadScene datasets; “eir.” denotes the corresponding existing image restoration preprocessing method used for each degradation setting, red indicates the best result, and blue indicates the second-best result.

Method	MFNet Dataset			DN-MSRS Dataset			RoadScene Dataset		
	SD	EN	MUSIQ	SD	EN	NIQE	SF	NIQE	BRISQUE
eir. + ReCoNet	41.654	5.161	29.299	41.525	4.463	8.631	10.312	4.785	37.775
eir. + PLAFusion	39.853	6.123	34.184	36.952	6.025	5.083	14.852	3.864	31.651
eir. + U2Fusion	33.945	5.741	34.255	28.812	4.609	7.185	18.006	4.215	34.577
eir. + MetaFusion	42.026	6.665	34.764	39.956	6.398	4.337	26.653	3.473	29.521
eir. + SemLA	32.622	5.982	33.526	30.654	5.323	5.882	12.252	3.988	32.253
eir. + MRFS	38.651	6.225	34.325	36.211	5.958	5.151	14.487	3.843	31.458
eir. + SAGE	42.882	6.568	35.253	41.957	6.453	4.525	16.543	3.625	30.053
eir. + DCEvo	43.553	6.755	35.525	43.055	6.554	4.254	17.726	3.522	29.254
OURS	44.025	6.689	35.852	43.522	6.661	4.053	17.701	3.381	28.958

**Table 4:** Comparison of model complexity and running efficiency of different methods on the VIS–IR task; red indicates the best result, and blue indicates the second-best result.

Models	Params (M)	FLOPs (G)	MSRS (FPS)	LLVIP (FPS)	RoadScene (FPS)
ReCoNet	0.441	10.81	12.82	12.35	11.9
PIAFusion	0.392	9.14	13.33	12.82	12.99
U2Fusion	1.095	28.92	2.92	2.72	2.85
MetaFusion	0.272	5.23	17.86	16.39	17.24
SemLA	0.793	18.74	8.77	8.26	8.47
MRFS	0.325	7.91	15.63	14.93	15.15
SAGE	1.171	31.62	6.9	6.62	6.76
DCEvo	1.362	36.86	6.37	6.02	6.17
OURS	0.346	8.04	15.87	15.38	14.08

**Table 5:** Quantitative results of the ablation study.

Setting	EN	SD	SCD	VIF	$Q^{AB/F}$
w/o $t_{GT}$	7.567	50.285	1.724	1.353	0.687
w/o SFAE	7.442	48.291	1.715	1.237	0.612
w/o SFRF	7.323	46.325	1.533	1.271	0.599
OURS	7.642	51.462	1.781	1.402	0.712

## Data Availability

This manuscript encompasses all data that were produced or examined throughout the course of this study. Accompanying scripts and computational methods integral to the data’s creation will be made available in due course.
